# Engaging GPs in insulin therapy initiation: a qualitative study evaluating a support program in the Belgian context

**DOI:** 10.1186/1471-2296-15-144

**Published:** 2014-08-21

**Authors:** Patricia Sunaert, Sara Willems, Luc Feyen, Hilde Bastiaens, Jan De Maeseneer, Lut Jenkins, Frank Nobels, Emmanuel Samyn, Marie Vandekerckhove, Johan Wens, An De Sutter

**Affiliations:** 1 Department of General Practice and Primary Health Care, Ghent University, De Pintelaan, 185, 9000 Ghent, Belgium; 2 Department of General Practice, Interdisciplinary Healthcare and Geriatrics, Antwerp University, Antwerp, Belgium; 3 Diabetes Project Aalst, Ghent, Belgium; 4 Department of Clinical and Lifespan Psychology & Department of Experimental and Applied Psychology, University of Brussels, Brussels, Belgium

**Keywords:** Type 2 diabetes, Insulin therapy, Primary care

## Abstract

**Background:**

A program supporting the initiation of insulin therapy in primary care was introduced in Belgium, as part of a larger quality improvement project on diabetes care. This paper reports on a study exploring factors influencing the engagement of general practitioners (GPs) in insulin therapy initiation (research question 1) and exploring factors relevant for future program development (research question 2).

**Methods:**

We have used semi-structured interviews to answer the first research question: two focus group interviews with GPs who had at least one patient in the insulin initiation program and 20 one-to-one interviews with GPs who were not regular users of the overall support program in the region. To explore factors relevant for future program development, the data from the GPs were triangulated with data obtained from individual interviews with patients (n = 10), the diabetes nurse educator (DNE) and the specialist involved in the program, and data extracted from meeting reports evaluating the insulin initiation support program.

**Results:**

We found differences between GPs engaged and those not engaged in insulin initiation in attitude, subjective norm and perceived behavioural control regarding insulin initiation. In general the support program was evaluated in a positive way by users of the program. Some aspects need further consideration: job boundaries between the DNE and GPs, job boundaries between GPs and specialists, protocol adherence and limited case load.

**Conclusion:**

The study shows that the transition of insulin initiation from secondary care to the primary care setting is a challenge. Although a support program addressing known barriers to insulin initiation was provided, a substantial number of GPs were reluctant to engage in this aspect of care. Important issues for future program development are: an interdisciplinary approach to job clarification, a dynamic approach to the integration of expertise in primary care and feedback on protocol adherence.

**Trial registration:**

ClinicalTrials.gov Identifier: NCT00824499

## Background

Type 2 diabetes (T2DM) is characterized by both insulin resistance and inadequate insulin secretion. As T2DM is a progressive disease many patients require insulin therapy at some point in time [1]. The number of T2DM patients on insulin therapy is rising due to the increasing prevalence of T2DM, the more stringent glycaemic targets and improved life expectancy among T2DM patients. In 2004 396,481 persons in Belgium were reimbursed at least one package of diabetes medication of whom 104,780 were prescribed insulin therapy; in 2010 this was respectively 489,559 and 136,753 [2].

Although insulin therapy is no longer a secondary care based activity in countries like the United Kingdom (UK) and the Netherlands, the involvement of general practitioners (GPs) is still limited in many other countries [3-5]. There is no evidence that the outcomes of insulin therapy are different when managed in primary care instead of in secondary care. However, there are pragmatic reasons to involve primary care in insulin therapy. First of all, many T2DM patients have more than one chronic condition and will benefit from a holistic approach [6,7]. Further, it is expected that involvement of primary care in insulin therapy will decrease the delay in treatment intensification. Furthermore, there are simple insulin regimens that are easy to start within primary care [8].

GPs in Belgium are encouraged to take up a more active role in insulin therapy since the introduction of a care pathway T2DM by the National Institute for Health and Disability Insurance (NIHDI) [9]. This paper reports the findings of a study we performed before the introduction of the care pathway T2DM in 2009. In this study we explored ways to improve care delivery for T2DM patients using a complex intervention targeting all T2DM patients and their care providers in a well-defined geographical region in Belgium (2003–2007) [10].

One of the interventions was the introduction of a support program for insulin initiation in primary care. At the end of the study period 56% (response rate 80%) of the GPs reported starting insulin therapy in practice, 33% of the GPs in the region had at least one patient in the program (unpublished data).

This paper reports on a study evaluating the support program at the end of the study period.

The paper addresses the following research questions:

1. Which factors influenced the engagement of GPs in insulin initiation?

2. What lessons can be learned for future program development?

## Methods

### Design

A qualitative study exploring factors influencing the engagement of GPs in insulin initiation in a context where a support program is provided and exploring factors relevant for future program development.

### Setting

The region counted 83 GPs (of whom 70% worked in a single-handed practice) for 76,826 inhabitants and two hospital based diabetes centres. Since 1987, hospitals in Belgium are financed by NIHDI to establish multidisciplinary diabetes centres. In these centres patients receive care from a diabetes team (diabetologist, diabetes nurse educator (DNE) and dietician) and obtain material for self-monitoring of blood glucose (SMBG) for free [11]. At the start of the study most GPs referred their patients to one of the two hospital diabetes centres once insulin was required.

### Characteristics of the support program

We developed a multifaceted support program addressing the main barriers to insulin initiation reported in the literature (Table 1) [12-15]. Participation of care providers (DNE, GPs, specialists, certified diabetes nurses (home nursing)) was solicited by means of study groups with the aim of translating the program to the local context [16]. This process resulted in an interdisciplinary protocol (http://www.diabetesprojectaalst.be). GPs were assigned a central role in the management of patients eligible for a once-daily insulin regimen. Starting up a basal insulin regimen represents a simple and effective way to start insulin therapy [8]. Once patients required a more complex scheme, referral to secondary care was advised. GPs were expected to discuss the option of insulin therapy with their patients when oral therapy failed (indication, benefits, and barriers) and to take care of the follow-up. Furthermore, GPs were advised to refer their patients to a DNE once insulin therapy was considered. During the study a well-trained DNE was engaged who operated from a community diabetes centre in the region. Less mobile patients were instructed at home. The service was free of charge for the patients and the DNE was paid a wage (breaking up with the usual fee-for-service system in the Belgian context). As such a flexible program, meeting the needs of the patients, could be offered. Patients also received SMBG material for free. Certified diabetes nurses were involved when patients needed (temporarily) support at home for administering insulin injections. Specialists were expected to coach the GPs, the DNE and home nurses. One specialist (FN) invested on average 4 hours a month to supervise the content of the program and to coach the DNE. To enhance self-confidence among GPs we organized two interactive workshops, one at the start of the study, and another one six months later, to familiarize with the proposed insulin regimen.

**Table 1:** Multifaceted support program addressing known barriers to insulin initiation

***Addressing patient barriers: misconceptions about insulin therapy, fear of injections, fear of hypoglycaemia, fear that insulin therapy will interfere with daily living, social stigma***	
•	Structured education program: patient education checklist addressing potential barriers and misconceptions, familiarising with what insulin is, guiding on when and who to call for problems, .…
•	Educational tools for patients: information sheets regarding SMBG, insulin administration, managing hypoglycemia; patient booklet; website [http://www.diabetesprojectaalst.be]
•	SMBG-material for free; devices corresponding with patient’s needs and capabilities.
***Addressing physician barriers: doubts about the benefit of insulin therapy, lack of knowledge, lack of familiarity, concerns about the risk of hypoglycaemia, concerns about patient compliance, time constraints, lack of staff support***	
•	Simplified treatment protocol starting with a once-daily insulin regime.
•	Provider education: interactive workshops (main topics: indications for insulin therapy in T2DM, starting up a once daily basal insulin regimen, insulin titration algorithm and collaboration modalities, website (http://www.diabetesprojectaalst.be), continuous feedback by DNE.
•	Coaching of GPs by specialists from the 2 hospital diabetes centres (e-mail, phone).
•	Team-working approach: interdisciplinary protocol with clear job descriptions.
•	Appointment of a well-trained diabetes nurse educator (DNE).
•	Formal mentoring of DNE by one of the specialists from the region.

### Data collection

We used semi-structured interviews to answer the first research question: two focus group interviews with GPs who had at least one patient in the insulin initiation program and 20 one-to-one interviews with GPs who were not regular users of the overall support program in the region. In order to explore factors relevant for future program development, the data from the GPs were triangulated with data obtained from one-to-one interviews with patients (n = 10), the DNE and the specialist involved in the program development, and data extracted from meeting reports evaluating the insulin initiation support program. All data were collected according to standardized procedures [17,18].

GPs who had at least one patient in the insulin initiation program during the first study year (n = 13) were invited to take part in a focus group meeting. Two GPs refused to participate, one because of time constraint, another because the interview was planned during the evening hours. Two GPs called off at the last moment for reasons of occupational activities (too busy in practice). A purposive sample of 20 GPs (degree of participation in the overall program, gender, age, practice type) was selected for one-to-one interviews. Five could not be interviewed: four GPs refused (two because of time constraint, two without a formal reason and one GP was abroad when the interviews were planned). They were replaced by GPs with a comparable profile. Patients who were initiated insulin therapy in the first study year and had a follow-up of at least one year in the community diabetes centre (n = 14) were eligible for the one-to-one interviews. Four could not be interviewed; one because of cognitive deterioration, three refused without a formal reason.

The focus group interviews were moderated (PS) and observed (LF) by members of the research team (Department of General Practice and Primary Health Care, Ghent University). Participating GPs knew both researchers from meetings in the region. The DNE participated in the focus group interviews in order to clarify some aspects of the support program. The one-to-one interviews (patients and GPs) were conducted by a trained interviewer (psychologist- MV) who was familiar with the program and the interview aims but was not involved in the program. Verbal informed consent was obtained from all interview participants. All participants were informed about the purpose of the interviews (evaluation of participants’ experiences with the introduction of a new service in the region) at the time of the invitation and at the start of the interview.

Focus group interviews and one-to-one interviews with patients took place at the community diabetes centre. One-to-one interviews with GPs took place at the GP’s practice. All interviews were audiotaped and transcribed verbatim. Data extraction from meeting reports was performed by PS.

### Data analysis

Data analysis of the different data sources followed the principles of content analysis and constant comparison and was guided by the research questions [19]. Data management was undertaken manually. In order to organize the data relevant for the first research question the theory of planned behaviour (TPB) was used [20]. This social cognition model has been widely used to predict individual behaviours, and has been one of the theories used most often when exploring determinants of professional behaviour. The theory states that an individual’s intention to perform a behaviour is the proximal predictor of behaviour. In turn intention is predicted by attitude (the degree to which a person has a favorable or unfavorable evaluation or appraisal of the behaviour in question), subjective norm (the perceived social pressure to perform or not to perform the behaviour) and perceived behavioural control (the perceived ease or difficulty of performing the behaviour).

Two researchers (LF, PS) performed the triangulation process guided by the triangulation protocol of Farmer to answer research question 2 [21]. Both researchers first analyzed the data independently (sorting, convergence coding, convergence assessment, completeness assessment). Next, consensus on the key themes emerging from the data was reached through discussion (researcher comparison). A summary of the triangulated results was sent for review to the members of the local steering group in the region. They agreed with the results.

### Ethical considerations

The study was approved by the Ethics Committee of the University of Ghent (Approval number 2004/253) and the Ethics Committee of the University of Antwerp (Approval number 12/07/2004).

## Results

Table 2 gives a summary of the characteristics of the interview participants.

**Table 2:** Background data of the interview participants

Focus group interviews with GPs (n = 9)	Mean age (SD): 50,8 (10,1; 31–68)
	[<40 years: 1; 40–50 years 3; 50–60 years: 4; > 60 years:1]
	Female: 1
	Single-handed practice: 5
One-to-one interviews with GPs (n = 20)	Mean age (SD): 48,2 (9,2; 31–65)
	[<40 years: 3; 40–50 years: 8; 50–60 years: 7; > 60 years:2]
	Female: 4
	Single-handed practice: 17
One-to-one interviews with patients (n = 10)	Mean age (SD): 67,1 (10,8; 54–82)
	[50–60 years: 3; 60–70 years: 2; >70 years 5]
	Female: 3
	Diabetes duration (SD): 11,7 (6,4; 5–25)
	HbA1c% (SD): 9,4* (1,3; 7,6-11,7); *79 mmmol/mol

### Factors influencing the engagement in insulin initiation (research question 1)

**Figure 1:**
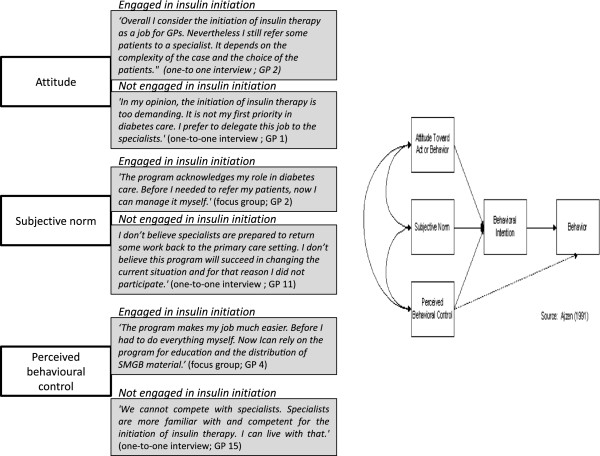
Quotes in relation to GPs’ engagement in insulin initiation (TPB).

We found differences between GPs engaged and those not engaged in insulin initiation in attitude, subjective norm and perceived behavioural control regarding insulin initiation. The findings are illustrated with some quotes in Figure 1.

### Attitude

For some GPs the transition of insulin initiation to the primary care setting increased job satisfaction. They were eager to be involved and their engagement was reinforced by the positive feedback of their patients. Others appreciated the opportunity to start insulin therapy for some patient groups, e.g. the elderly. Their engagement depended on the complexity of the case and the choice of the patients. Some GPs felt no need for change. Most of them expected no added value (for themselves or their patients) of being involved in insulin initiation. On the contrary, they perceived this aspect of care as too demanding. They preferred to refer their patients to the specialist once insulin therapy was required and did not intend to change their habits.

### Subjective norm

Some GPs perceived the transition of insulin initiation to the primary care setting as an acknowledgment by health policy of their role in diabetes care. The feeling of recognition of their role in diabetes care was reinforced by the positive attitude of specialists regarding the program. Others were suspicious regarding the outcome of the study and the motives of health policy. They did not believe that specialists would agree with the transition of this aspect of care to the primary care setting. For this reason they did not engage in the program.

### Perceived behavioural control

For some GPs, the launch of the program was the impetus to take up this aspect of care. They appreciated the opportunities to become more knowledgeable regarding insulin initiation. The support program gave them the confidence to engage in insulin initiation. As a result they stressed on the importance of the continuity of the program for their involvement in insulin initiation. However, a substantial number of GPs remained insecure regarding insulin initiation. In particular, the increasing complexity of insulin regimens made some GPs reluctant to engage in insulin therapy. Most of them did not attend the workshops since they did not intend to engage in insulin initiation. Further, the risk for hypoglycaemia was an important reason why some GPs perceived this aspect of care as too risky to be involved in. They felt not well enough equipped in comparison with specialists to handle this problem.

### Factors relevant for future program development (research question 2)

In general the support program was evaluated in a positive way by users of the program (patients and GPs). The main aspects related to program satisfaction are discussed and illustrated with some quotes (Table 3). Some aspects need to be taken into account in future program developments: job boundaries between the DNE and GPs, job boundaries between GPs and specialists and protocol adherence.

**Table 3:** Main themes and subthemes in relation to future program development

**Aspects related to program satisfaction**	
***Patient views***	*‘It is easier to start in primary care. Our GP lives across the street. He’s known us for years.’* (interview patients; P2) *‘The DNE has the time to give us information. She explained everything to me; why I have to use insulin, how I should use it, … it is really nice.’* (interview patients; P 3) *“They” (DNE, GP) work together very well. On one occasion my results were not good. The DNE phoned my GP and they agreed on changing my therapy.’* (interview patients; P 9)
- GP is in charge	
- information	
- emotional support	
- time	
- flexibility	
- free of charge	
- collaboration between DNE and GP	
***GP views***	*‘The workshop was very good. The information was to the point, very clear.’* (focus group; GP 2) *‘For most patients insulin therapy is still the last thing they want to start with. They have no problems with an extra tablet, but starting insulin therapy is still an enormous threshold to overcome. Although, once they are on insulin most of them have no problem with it.’* (focus group; GP 9) *‘The DNE has the competence and the time to give information to the patient. When we engage ourselves in insulin therapy, we have to do it well. With the support of the DNE I feel comfortable.’* (focus group GP 8)
- information in workshops	
- protocol	
- support in primary care	
- overcoming patients’ barriers	
- patients are satisfied	
- structured approach by DNE	
- communication with DNE	
- coaching by the specialist	
**Aspects for consideration in future program development**	
***Job boundaries between DNE and GPs***	*‘We were not used to this kind of service in primary care. We need time to learn how we can work together.’* (focus group; GP 3) *‘I see my patient every three months. In between the DNE is responsible for adjusting insulin doses. She communicates with the patient regarding this topic. I feel relieved that I can delegate this job to her.’* (focus group; GP 4)
- new function in primary care	
- fear to lose the patient	
- control over therapy	
- limited tradition of collaboration	
***Job boundaries between GPs and specialists***	*‘Patients in the program receive the advice to consult the specialist once they require two or more injections a day. This has prevented me from referring patients to the support program.’*(one-to-one interview; GP 3*)*
- limitation to once daily insulin regimen	
- engagement of specialists	
***Protocol adherence***	*‘When insulin therapy is initiated, patients always do more measurements. There is ’the fear of hypoglycaemia’. I suppose this is normal.’* (focus group, GP 8) *‘I like to have a glycaemic value during the day. It reassures me.‘* (focus group; GP 1)
- postponement of dose titration	
- fear of hypoglycaemia	
- treating the regimen as a complex one	
- limited engagement of patients in dose adjustment	

### Aspects related to program satisfaction

#### Patient views

Patients were very positive regarding the offer and hoped the program would continue in the future. They appreciated the fact that they could be helped in primary care and some mentioned that the opportunity to start insulin therapy with their GP decreased the threshold to insulin therapy. Most patients expressed that the transition to insulin therapy was a difficult step to take and that the DNE helped them with making this transition. Some patients especially valued the emotional support and the possibility to have an extra session if needed (flexibility of the offer). The fact that the service (education, SMBG-material) was free of charge was very important for some patients. The close collaboration between the DNE and the GP enhanced patients’ confidence in the program.

#### GP views

GPs valued the information given in the workshops and the opportunity for feedback from the DNE and the specialists. All GPs evaluated the support by the DNE in a positive way. Most GPs reported that patients try to postpone insulin therapy as long as possible. GPs experienced that the DNE was able to overcome barriers in most cases. Some GPs experienced that gaining more confidence in insulin therapy had also a positive effect on patients’ perception of insulin therapy.

### Aspects for further consideration

#### Job boundaries between GPs and the DNE

Since the DNE was a new job in primary care, uncertainty about job boundaries rose among GPs. Some GPs feared further fragmentation of care and losing control over therapy. However, once they had a patient in the program most GPs valued the specific competence of the DNE. Gradually, the role of the DNE evolved to an expert role and GPs could rely on her advice for patient related problems. The DNE noticed that she often had to take the initiative for consultation (questionnaire DNE, meeting reports). GPs acknowledged this and remarked they needed time to get used to this kind of service.

Some uncertainty remained regarding the responsibility for the adjustment of insulin doses. Some GPs - often the most experienced ones - were happy to delegate this job to the DNE. Others preferred to keep control of this part of the job and made arrangements with patients, e.g. a phone call every week.

The DNE tried to overcome uncertainty regarding job boundaries by consulting GPs on a regular basis (e-mail, phone), e.g. asking for consent before adjusting insulin therapy. Although GPs appreciated this way of working it is probably not feasible to continue this strategy when the workload increases (questionnaire DNE, meeting reports). The DNE emphasized that, in order to take up an expert role in the future, it is crucial that the case load for a DNE is high enough and that opportunities for continuous education are provided (questionnaire DNE).

#### Job boundaries between GPs and specialists

The readiness to involve GPs in insulin therapy varied among specialists in the region. Some specialists hesitated to engage GPs in insulin therapy, others referred patients (eligible for a once-daily insulin regimen) back to primary care. Since at the start of the study most GPs were not involved in insulin initiation, representatives of specialists and GPs agreed (study groups) to limit the role of GPs to a once-daily insulin regimen. For some GPs this compromise was a barrier to engage in the program. They perceived the restriction as an indication that specialists were not willing to share insulin therapy with primary care.

### Protocol adherence

Most GPs reported that the once-daily insulin regimen was very practical to use. However most of them seldom consulted the proposed protocol after the introduction in the workshop. As a consequence, the DNE experienced that some GPs did not respect the guidelines regarding insulin dose adjustment and SMBG control. Some patients remained on an insufficient insulin dose too long; others were shifted to a complex insulin scheme too fast (questionnaire DNE; meeting reports). The risk for hypoglycaemia remained a point of concern among the majority of GPs. For this reason most GPs reported asking patients to control glycaemic status more often than advised in the protocol. According to the DNE this approach risks to increase fear for hypoglycemia among patients. The appropriate way to deal with this concern is to provide adequate hypoglycaemia education (questionnaire DNE). Since a once-daily insulin regimen was barely used in the region before the study started, specialists had a learning curve too. They tended to handle the simple regimen as a complex one, advising insulin (and oral medication) dose adjustments on glucose curves instead of on fasting blood glycaemia levels in the morning (meeting reports).

## Discussion

The study shows that the transition of insulin initiation from secondary care to the primary care setting is a challenge. Although a support program addressing known barriers to insulin initiation was provided, a substantial number of GPs were reluctant to engage in this aspect of care. The most striking differences between GPs engaged and those not engaged in insulin initiation were observed on the attitude towards the involvement in insulin therapy, the perceived pressure to involve themselves in insulin therapy and the perceived self-confidence. Overall, the support provided in the program was evaluated in a positive way by users of the program (both by patients and GPs). Most patients attending the program were able to overcome barriers and appreciated being helped in the primary care setting.

### Implications for future program development

#### An interdisciplinary approach to role clarification

At the start of the study there was no clarity on the role of GPs regarding insulin therapy. This can partly explain the difference in attitude among GPs regarding their role in insulin therapy initiation and in the perceived pressure to engage themselves in insulin therapy. To overcome this ambiguity we assigned GPs a central role in the initiation of a once-daily insulin regimen (in consultation with the other care providers involved). The problem of role ambiguity is not limited to our context [22]. What makes the problem more prominent in our study is the hesitation of health policy leaders to reinforce the role of GPs in the health care system [10]. The study was conducted in a context where many GPs felt insecure and frustrated about their role in health care. They felt somehow powerless as they could not compete with the expertise and service delivered in secondary care [10]. This climate hampered the uptake of insulin initiation therapy among GPs. Further, the tradition of interprofessional collaboration was still limited. Our study was one of the first in the region to start a process that led to an interdisciplinary care protocol and as such contributed to a collaborative way of working [10].

#### A dynamic approach to integration of expertise in primary care

During the first year of the study 80 patients in the region received a first prescription for insulin therapy, 20 of whom attended the support program in primary care (unpublished data). Given the relatively small and mostly single-handed practices in Belgium (GP to population rate of 1:925) the number of patients starting insulin therapy (one patient per GP during the period of one year on average) limits the possibility for GPs to gain confidence. However, as observed in our study, the collaboration with an experienced DNE and the opportunity to receive feedback by specialists can reassure GPs to engage in this aspect of care and to gain confidence over time. As such the DNE is likely to be pivotal for a successful shift of insulin initiation to the primary care setting. In our study, support was provided by a DNE operating in a community diabetes centre. She was in charge of all patients starting insulin therapy in the program and gained confidence and expertise when her case load gradually increased. By consequence, her role extended to an expert role.

During the study period the DNE was coached by a specialist and further collaboration with specialists was solicited in the future (explicitly asked by the DNE). This raises questions on responsibilities when insulin therapy is initiated in the primary care setting. Who is responsible for the DNE: the GP by which the patient is referred or the specialist (coach)? What are the responsibilities of the DNE? Similar questions were raised in a recent study in Australia [23]. Our study suggests that clarifying responsibilities requires a dynamic approach with the emphasis on what the patient needs rather than on organizational boundaries [24]. For example, according to the protocol patients had to break up their relationship with the DNE in primary care once they required a more complex insulin scheme. This was in contrast with the perspective of continuity and the patients’ preference.

A dynamic approach should result in shared responsibilities that can vary in function of the complexity of the case, the experience of the individual GP and the available support in practice.

#### Feedback on protocol adherence

The importance of protocol adherence for the effectiveness of the proposed insulin regimen became apparent in the course of the study. The DNE reported problems in relation to the adjustment of insulin doses and SMBG control in several cases. The delegation of some tasks of the protocol to the patient or the DNE is probably the most suitable solution for this problem [25]. Data from clinical trials show that a patient-directed approach to insulin dose titration is a safe and effective alternative to a physician-directed approach [26]. Further, fear of hypoglycaemia was an important reason to deviate from the protocol among patients and GPs. The DNE emphasized that the most important way to handle the problem of hypoglycaemia is repeated education, which should empower patients to deal with the problem.

### Comparison with existing literature

The literature reports extensively on barriers to insulin therapy among patients and care providers [12-15]. Our study evaluates a program supporting the transition of insulin initiation to the primary care setting. To our knowledge research on this subject is scarce.

Our findings are consistent with the literature on care innovation [27]. As most GPs were used to refer their patients to secondary care, GPs were asked to change their routines. Changing routines takes time and needs repeated efforts, targeting different levels of the adoption process [28]. A recent study evaluating a support program utilizing diabetes experts and community retail pharmacists to enhance insulin prescribing in family practice came to the same conclusion [29].

Our study also confirms literature findings about patient barriers towards insulin therapy. Patients are still trying to postpone insulin therapy as long as possible. Overcoming GP barriers to insulin initiation is a prerequisite to tackle reluctance among patients [30]. In our study GPs experienced that gaining more confidence in insulin therapy had also a positive effect on patients’ perception of insulin therapy.

### Strengths and limitations of the study

An important strength of our study is that we were able to include findings not only about GPs intending to engage, but also about those who were not. The group of non-participants is often not represented in evaluation studies. The use of a well-established psychological model (TPB) in the reflection on the data led to a deeper insight into factors that influence engagement of GPs in insulin initiation. The triangulation of findings from different viewpoints (patients, GPs, DNE, specialist) contributes to the internal validity of the findings regarding the program evaluation. However, the findings on program evaluation are only representing the view of users of the program. As non-users had no experience with the program we did not ask them questions about the content of the program. Our study findings cannot claim generalizability. However, we aimed for transferability of the results by giving clear information regarding the context and the setting in which the data were collected and interpreted. The relevance of the study results for the current Belgian situation is clear as it became apparent that the engagement of GPs in insulin initiation, as encouraged by the care pathway T2DM (a new national initiative of the NIHDI started in September 2009), was not self-evident. Similar problems as during our study occurred and the engagement of GPs appeared to be a critical issue in the success of the pathway. As such our study contributes to a more in-depth understanding of the factors influencing the engagement of GPs in insulin initiation (research question 1) and helps to adapt the support program by sharing lessons learned (research question 2). The relevance of the findings for other settings will differ in relation to the way insulin initiation is organized currently and in relation to the strength of the primary care system. For settings planning to involve primary care in insulin initiation our findings will help to develop a support program and to anticipate some of the barriers we experienced. As our findings suggest one can expect that setting up such a program will encounter less barriers in a primary health care setting where the role of GPs is clearly defined, support staff is available and collaboration between primary and secondary care is well established [31]. For settings where support for insulin initiation is already provided in primary care our findings can be of interest in exploring why there is still a delay in starting up insulin therapy when metabolic control is not adequate [32].

## Conclusion

The study shows that the transition of insulin initiation from secondary care to the primary care setting is a challenge. Although a support program addressing known barriers to insulin initiation was provided, a substantial number of GPs were reluctant to engage in this aspect of care. Important issues for future program development are: an interdisciplinary approach to job clarification, a dynamic approach to the integration of expertise in primary care and feedback on protocol adherence.

## Competing interests

The authors state that they have no conflict of interests.

## Authors’ contributions

PS, SW, LF, HB, JD, FN, JW and AD were involved in the conception and design of the study. PS, LF, HB and MV were involved in the acquisition and analysis of the data. All authors were involved in the interpretation and discussion on the data and the regular revision of the drafts. All authors read and approved the final manuscript.

## Pre-publication history

The pre-publication history for this paper can be accessed here:

http://www.biomedcentral.com/1471-2296/15/144/prepub
